# Dataset on differential gene expression analysis for splenic transcriptome profiling and the transcripts related to six immune pathways in grass carp

**DOI:** 10.1016/j.dib.2016.12.048

**Published:** 2016-12-29

**Authors:** Guoxi Li, Yinli Zhao, Jie Wang, Bianzhi Liu, Xiangli Sun, Shuang Guo, Jianxin Feng

**Affiliations:** a College of Animal Science and Veterinary Medicine, Henan Agricultural University, Zheng zhou, Henan Province 450002, PR China; b College of Biological Engineering, Henan University of Technology, Zheng zhou, Henan Province 450001, PR China; c Laboratory of Aquaculture and genetic breeding, Henan Academy of Fishery Science, Zheng zhou, Henan Province 450044, PR China

## Abstract

The data presented in this paper are related to the research article entitled “Transcriptome profiling of developing spleen tissue and discovery of immune-related genes in grass carp (*Ctenopharyngodon idella*)” (Li et al. 2016) [1]. Please refer to this article for interpretation of the data. Data provided in this submission are comprised of the expression levels of unigenes, significantly differentially expressed genes(DEGs), significant enrichment GO term and KEGG pathway of DEGs, and information of the transcripts assigned to six immune pathways.

**Specifications Table**

**Table:** 

Subject area	Biology
More specific subject area	Transcriptomics
Type of data	Excel file
How data was acquired	High-throughput RNA-sequencing
Data format	Analyzed
Experimental factors	All animal experiments were performed as described previously [1]. The spleen samples were collected from one-year (cis1) and three-year-old (cis3) grass carp *C. idella.*
Experimental features	cDNA libraries were constructed using a TruseqTM RNA sample prep Kit (Illumina). The paired-end cDNA library was sequenced on an Illumina HiSeq4000. Differential gene expression analysis was performed as described in [1]. KEGG functional classification was performed as described in [2].
Data source location	N/A
Data accessibility	Analyzed datasets are contained within this article. Raw data was deposited in the NCBI database Sequence Read Archive under accession number SRP078553.

**Value of the data**

• The data provide the significantly differentially expressed genes in the grass carps between one-year and three-years of age, which may be used for future research on the molecular mechanisms of the spleen׳s late development of the fish.
• The data contributes to the understanding of transcriptome profiling of developing spleen tissue in grass carp.
• The transcripts assigned to six immune pathways in this dataset could be applied in discovery of immune-related genes in grass carp and also serve as a reference for research on the immune system in the other closely related species.

## 1 Data

The transcriptome sequencing from the spleen tissue of one-year-old (cis1) and three-year-old (cis3) grass carp was performed using Illumina paired-end sequencing technology. The dataset of this paper comprises ten data files that were generated from the KEGG annotation analysis and differential gene expression analysis of the transcriptome sequence above. The unigene expression levels are presented in File 1. The significantly differentially expressed unigenes between the cis1 and cis3 library are presented in File 2. The significant enrichment GO terms and KEGG pathways of DEGs are presented in Files 3 and 4, respectively. Information of the transcripts involved in the T-cell receptor signaling pathway, B-cell receptor signaling pathway, Toll-like receptor signaling pathway, antigen-processing and presentation, complement cascades, and chemokine signaling pathway are summarized on six files from Files 5 to 10, respectively.

## 2 Experimental design, materials and methods

### 2.1 Experimental animals

The experimental animals were the cultivated grass carp *Ctenopharyngodon idella.* The spleen tissues were collected from six individuals at each developmental stage.

### 2.2 cDNA library construction and sequencing

The cDNA library was constructed by using Truseq^™^ RNA sample prep Kit (Illumina) and sequenced on an Illumina HiSeq4000 sequencing platform that generated paired-end reads of 151 nucleotides. The detailed description can be seen in Ref. [1].

### 2.3 Differential gene expression analysis

Differentially expressed genes were identified by using the methods as described in Ref. [1]. Gene expression levels were calculated using the fragments per kilo bases per million mapped reads (FPKM) method[3].The settings ‘‘corrected *P*-value ≤0.05’’ and ‘‘absolute value of log 2 ratio ≤1’’ were used as thresholds for judging significant differences in transcript expression.

## Footnotes

Appendix A Supplementary data associated with this paper can be found in the online version at http://dx.doi.org/10.1016/j.dib.2016.12.048.
