# Supplementary material for: Dataset on differential gene expression analysis for splenic transcriptome profiling and the transcripts related to six immune pathways in grass carp
Source: Data Brief. 2016 Dec 29;10:598–600. doi: 10.1016/j.dib.2016.12.048 (PMC5226849; doi:10.1016/j.dib.2016.12.048)
Supplement: Supplementary file 1 — Supplementary material [file mmc11.pdf]

Article reference: DIB_DIB-D-16-00935
Article title: Dataset on differential gene expression analysis for splenic transcriptome profiling and the transcripts related to six immune pathways in grass carp
To be published in: Data in Brief

All the authors do the statement below.

Conflict of interest: The authors declare no conflict of interest

Signature: **Guoxi Li Yinli Zhao Jie Wang Bianzhi Liu**

Xiangli sun Shuang Guo Jianxin Feng

Date: January10, 2017
